# Extracellular vesicular miRNA expression is not a proxy for skeletal muscle miRNA expression in males and females following acute, moderate intensity exercise

**DOI:** 10.14814/phy2.14520

**Published:** 2020-08-18

**Authors:** Jessica L. Silver, Sarah E. Alexander, Hayley T. Dillon, Séverine Lamon, Glenn D. Wadley

**Affiliations:** ^1^ Institute for Physical Activity and Nutrition (IPAN) School of Exercise and Nutrition Sciences Deakin University Geelong VIC Australia

**Keywords:** endurance exercise, extracellular vesicle, microRNA, sex, skeletal muscle

## Abstract

Skeletal muscle and extracellular vesicle (EV) miRNA expression increases following acute endurance exercise. However, research to date has only been performed in males. The aim of this study was to describe the expression levels of a subset of miRNAs in EVs following acute exercise and compare it to skeletal muscle miRNA expression. Twelve males (age 22.9 ± 2.6 years, mean ± *SD*) and eight females (age 23.0 ± 3.4 years) cycled for 60 min at 70% VO_2_peak. Muscle biopsies and blood samples were collected at rest, immediately after and 3 hr after exercise. Acute exercise did not significantly alter the expression of miR‐1, miR‐16, miR‐23b and miR‐133a/b in EVs in males and females combined. There were no correlations between EV and skeletal muscle miRNA expression in any of the measured species at any time point. Exploratory analysis revealed differential miRNA responses to exercise between males and females. In males, a weak negative correlation was observed between skeletal muscle and EV miR‐16 expression immediately following exercise; however, the physiological relevance of this correlation is unknown. Additionally, when compared with values at rest, male skeletal muscle miR‐16 expression significantly increased immediately following exercise, whereas miR‐133a expression significantly decreased 3 hr post exercise. Our findings suggest that miRNAs isolated from EVs are not a proxy for skeletal muscle miRNA content. Our exploratory data is the first known evidence of sex‐specific differences in the miRNA response to an acute bout of endurance exercise, particularly for miRNA species implicated in mitochondrial metabolism and angiogenesis.

## Graphical abstract

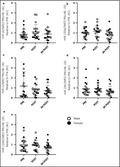

## 1 INTRODUCTION

Skeletal muscle is a dynamic tissue that rapidly adapts to molecular and environment stimuli, such as the metabolic stress associated with endurance exercise (Frontera & Ochala, 2015). Within skeletal muscle, endurance exercise upregulates mitochondrial biogenesis and increases the capacity of contracting muscle to oxidise carbohydrate and lipid substrates (Holloszy & Coyle, 1984; Little, Safdar, Benton, & Wright, 2011). When sustained over time, endurance exercise training promotes systemic adaptations that positively affect the cardiovascular and endocrine systems, whilst reducing the risk of metabolic conditions (Russell, Foletta, Snow, & Wadley, 2014). The intracellular transcriptional networks governing endurance exercise adaptations are well‐established; for example, *PGC‐1α*, an important regulator of skeletal muscle mitochondrial biogenesis, is transiently upregulated in response to acute endurance exercise (Puigserver et al., 1998). However, the mechanisms by which these skeletal muscle adaptations translate to systemic adaptations in other metabolic tissues (i.e., kidney, liver, cardiac muscle) are less clear (Little et al., 2011). Extracellular vesicles (EVs) may play a messenger‐like role in whole‐body signaling mechanisms by facilitating the transport of peptides and nucleic acids between tissues, which may then influence cell structure and function (Emanueli, Shearn, Angelini, & Sahoo, 2015; Guay & Regazzi, 2017; Safdar, Saleem, & Tarnopolsky, 2016; Valadi et al., 2007; Zhang et al., 2015). However, the role and regulation of EVs and their content in response to exercise is only starting to be investigated (D'Souza et al., 2018; Whitham et al., 2018).

Recent studies indicate that endurance exercise increases EV abundance within the circulation (Frühbeis, Helmig, Tug, Simon, & Krämer‐Albers, 2015; Guescini et al., 2015; Whitham et al., 2018). Although the specific stimuli triggering EV release following endurance exercise are unknown, increased intracellular calcium concentration (Savina, Fader, Damiani, & Colombo, 2005), potassium activation of neurons (Urbanelli et al., 2013) and mechanical stress may play important roles (Beninson & Fleshner, 2014; Guay & Regazzi, 2017; Whitham et al., 2018). EV content, or cargo, includes peptides (Ellingsgaard et al., 2011; Florholmen et al., 2006; Pedersen, 2007; Pedersen & Fischer, 2007; Seldin et al., 2013; Wallenius et al., 2002), cytokines and RNA molecules (Safdar, Abadi, Akhtar, Hettinga, & Tarnopolsky, 2009) which are also altered in response to endurance exercise (Whitham et al., 2018). Of particular interest are miRNAs, a class of noncoding RNAs (ncRNAs) that posttranscriptionally silence gene expression (Bartel, 2004). MiRNAs are differentially expressed within EVs following high intensity interval exercise (HIIE; D'Souza et al., 2018), and pose one possible mechanism by which exercise‐induced adaptations may be communicated throughout the body. Similarly, plasma miRNA expression is also altered following continuous, moderate intensity exercise. Studies so far have mostly investigated the circulatory miRNA profile in response to exercise, typically extracting miRNAs from whole plasma. EVs represent a small proportion of whole plasma content, and may therefore comprise a relatively dilute miRNA fraction when compared with circulating miRNAs (Arroyo et al., 2011; Chevillet et al., 2014). If EVs are released from contracting skeletal muscle, the miRNA profile within EVs may represent the miRNA profile within skeletal muscle, which could theoretically be used as a proxy measure of the skeletal muscle miRNA. In practice, this may reduce the need for invasive muscle biopsy procedures. Thus far, few studies have examined the relationship between skeletal muscle and EV miRNA expression (D'Souza et al., 2018). Furthermore, no studies have examined this relationship in response to continuous, moderate intensity exercise. Therefore, the aim of this study was to describe EV miRNA expression following an acute bout of endurance cycling and to compare it to skeletal muscle miRNA expression.

## 2 METHODS

### 2.1 Participant recruitment

This study was approved by Deakin University Human Research Ethics Committee (DUHREC 2014‐096) and conforms to the *Declaration of Helsinki*. Written, informed consent was obtained from all participants before commencing exercise trials and sampling procedures. Twelve healthy males (age 22.9 ± 2.6 years; mean ± *SD*) and eight healthy females (age 23.0 ± 3.4 years) were recruited. Participant anthropometrics are summarized in Table 1.

**Table 1:** Participant anthropometrics (mean ± *SD*). Male and female cohorts were matched for age, BMI and relative VO_2_peak. All participants completed the exercise protocol at approximately 70% VO_2_peak

	Males (*n* = 12)	Females (*n* = 8)
Age, year	22.9 ± 2.6	23.0 ± 3.4
Mass, kg	75.9 ± 9.0	65.9 ± 7.7*
BMI, kg/m^2^	24.6 ± 3.1	24.5 ± 2.5
VO_2_peak, ml min^−1^ kg^−1^	44.1 ± 8.7	40.7 ± 4.8
Relative workload, % VO_2_peak	68.3 ± 4.8	68.0 ± 4.4
Power at 70% VO_2_peak, W	133 ± 26	102 ± 13*
RER at 70% VO_2_peak	0.88 ± 0.05	0.80 ± 0.02*

* *p* < .05 (between groups).

Participants who were smokers, had a family history of heart disease or a relative peak oxygen consumption (VO_2_peak) of >55 or >50 ml kg^−1^ min^−1^ for males and females respectively, were excluded. Females not taking the oral contraceptive pill (OCP) were excluded from the study. Prior to testing, females completed a menstrual calendar (Knowles et al., 2019). Females completed all testing outside of the late‐follicular phase of the menstrual cycle (days 7–14), during which the ratio of oestrogen to progesterone peaks and the largest differences in performance are observed (Oosthuyse & Bosch, 2010).

### 2.2 Peak oxygen consumption

Participants reported to the laboratory, having performed no moderate‐vigorous exercise in the preceding 48 hr and underwent a maximal incremental exercise test on an electronically braked cycle‐ergometer (Lode, Groningen, the Netherlands) to determine VO_2_peak. Participants cycled at a self‐selected cadence throughout the test. Males and females began cycling at 75 W and 50 W, respectively, and increased by 50 W every three minutes for nine minutes. Thereafter, the workload increased by 25 W every minute until volitional fatigue. Individual VO_2_peak was calculated using data from expired gases collected every 15 s using an Innocur breath‐by‐breath metabolic system (Innovision, Glammsbjerg, Denmark). VO_2_peak was defined as the highest VO_2_ recorded over a 30 s period, at the point at which ΔVO_2_ < 2.1 ml min^−1^ kg^−1^ (Riebe, Ehrman, Liguori, & Magal, 2018; Taylor, Buskirk, & Henschel, 1955).

### 2.3 Exercise protocol

A wash‐out period of at least 48 hr separated the first and second trial to isolate the effects of the endurance exercise protocol and to eliminate any carry‐over effects of prior exercise. Participants reported to the laboratory at 7:00 a.m., having consumed no food since 9:30 p.m. the previous evening and no alcoholic or caffeinated beverages for 48 hr. Participants had access to water ad libitum. Participants undertook a 5‐min warm‐up at 50 W, before cycling for 60 min at a self‐selected cadence at the workload corresponding to 70% VO_2_peak, as described previously (Russell et al., 2013). VO_2_ was monitored intermittently throughout the exercise protocol to ensure adherence to the workload corresponding to 70% VO_2_peak. Participants then remained in a relaxed, supine position for 3 hr immediately following the exercise protocol.

### 2.4 Blood sampling

Prior to exercise, a 20‐gauge cannula was inserted into the ante‐cubital vein and blood samples collected in 4 ml vacutainer tubes containing 7.2 mg K2 EDTA (Becton Dickinson, Franklin Lakes, NJ, USA). Blood samples were collected from the ante‐cubital vein at rest (PRE), immediately after exercise (POST) and after 3 hr of passive recovery (3H POST). All blood samples were immediately centrifuged at 1,500 *g* for 10 min at 4°C. Plasma was stored at −80°C until further use.

### 2.5 Extracellular vesicle isolation from plasma

Extracellular vesicle isolation was carried out as described previously (Whitham et al., 2018). Briefly, frozen plasma was defrosted on ice, then centrifuged at 3,200 *g* for 20 min at 4°C to remove particulate matter. One ml of precleared plasma was diluted in equal volumes of ice‐cold phosphate buffered saline (PBS) and centrifuged for 1 hr at 20,000 *g* at 4°C. The supernatant was discarded and the pellet resuspended in 1 ml of PBS. The pellet was then spun again at 20,000 *g* for 1 hr at 4°C. The supernatant was removed and the pellet was resuspended in 110 µl of PBS. To remove all traces of potentially contaminating extra‐EV RNA, the isolated fraction was treated with an RNase‐A solution containing 0.4 mg/ml RNase‐A and Buffer P1 (Qiagen, Chadstone, VIC, Australia) as described previously (Barrey et al., 2011). RNase‐A activity was stopped by adding 5 μl of proteinase‐K (600 mAU/ml; Qiagen Inc.) and inverting the tube briefly. The solution was centrifuged at 8,000 *g* for 10 min at 4°C. The supernatant was aspirated, and the EV pellet was washed twice with 100 µl of PBS, before centrifugation at 3,000 *g* for 2 min at 4°C between washes.

### 2.6 Muscle biopsies

Skeletal muscle samples were obtained via muscle biopsy using the percutaneous muscle biopsy technique with a Bergstrom needle, modified to include suction (Bergstrom, 1962; Evans, Phinney, & Young, 1982). Briefly, the skin was anesthetized with 1% Xylocaine, and incisions were made through the skin and muscle fascia. Three muscle biopsies were obtained from the *vastus lateralis* of the same thigh, from three separate incisions approximately 20 mm apart. Muscle biopsies were taken at rest (PRE), immediately after exercise (POST) and after 3 hr of passive recovery (3H POST). Skeletal muscle samples were snap frozen and stored in liquid nitrogen until further analysis.

### 2.7 Skeletal muscle RNA extraction

Approximately 15–20 mg of skeletal muscle was homogenized with 600 μl Tri‐Reagent Solution (Ambion Inc., Austin, TX, USA) and 650–800 mg zirconia/silica beads (BioSpec Products, Bartlesville, OK, USA) in a MagNA Lyser for 30 s at 6,500 *g*. The lysate was transferred to a fresh tube and centrifuged at 13,000 *g* for 10 min at 4°C. The supernatant was transferred to a new tube and used for subsequent RNA extraction, modified to include half volumes of 1‐bromo‐3‐chloropropane in place of chloroform, according to the manufacturer's instructions. Skeletal muscle RNA was quantified using the Nanodrop 1000 Spectrophotometer. All samples returned A260/280 > 2.0 and A260/230 > 1.8.

### 2.8 EV RNA extraction

**Figure 1:**
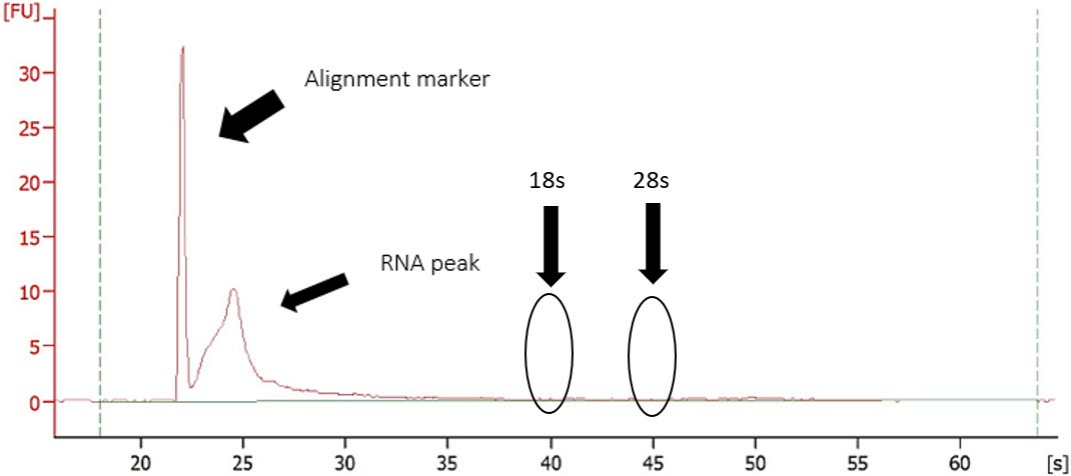
Representative electropherogram from the Agilent Picokit 6000. The graph shows good quality RNA, with a single peak, and no *indication of contamination by ribosomal 18s or 28s. The single peak shows a nucleotide length between 20 and 200 nt, indicative of small RNA*

An enriched small RNA fraction was extracted from isolated EVs using the miRNeasy Mini Kit, modified to include half volumes of 1‐bromo‐3‐chloropropane in place of chloroform, with on‐column DNase‐I digest (Qiagen Inc.) as per the manufacturer's instructions. EV RNA was eluted in 30 µl of ultra‐pure nuclease‐free water. EV RNA was quantified using the RNA 6000 Pico Kit (Agilent Technologies, Mulgrave, VIC, Australia) on an Agilent 2100 Bioanalyzer, according to the manufacturer's instructions. All samples included in the analysis were above the lower detection threshold (50 pg) of the assay. The resulting electropherogram for EV samples showed a single peak in the region of 25–200 nt, which is indicative of small RNAs. In the EV samples, there were no traces of 18s or 28s ribosomal RNA, indicating no contamination from cellular RNA (Figure 1). The resulting electropherogram (Figure 1) is very similar to that of a previous study that used the Agilent Bioanalyser to assess RNA from EVs (Helwa et al., 2017). Helwa et al. (Helwa et al., 2017) confirmed the presence of EVs in human plasma via nanoparticle tracking analysis, immunoblotting and transmission electron microscopy and used the same RNA quantification methods as in this study. Similar to the results of this study, RNA from EVs show a single peak at 25–20 nt, with no indication of 18s or 28s peaks, suggesting that we only assessed EV RNA in this study.

### 2.9 Reverse transcription

Skeletal muscle (50 ng) and EV RNA (500 pg) was reverse transcribed using the Taqman MicroRNA Reverse Transcription Kit and Taqman miRNA Assays (5X primers; hsa‐miR‐1, ‐16, ‐23b and ‐133a/b; Life Technologies, Mulgrave, VIC, Australia) according to the manufacturer's protocol with modifications to amplify multiple miRNAs (previously validated by (Le Carre, Lamon, & Leger, 2014)). For EV RNA samples, 0.1 µl/sample of spike‐in cel‐miR‐39 was added to the master mix as an exogenous control (Guescini et al., 2015; Nielsen, Åkerström, et al., 2014). The reverse transcription protocol consisted of 30 min at 16°C, 30 min at 42°C, and 5 min at 85°C. Following cDNA synthesis, skeletal muscle and EV cDNA was diluted 12.5× and 3×, respectively.

### 2.10 RT‐qPCR

MiRNA expression was assessed via RT‐qPCR using an AriaMX Thermal Cycler (Agilent Technologies). MiRNA expression was measured using the Taqman Universal Master Mix II, no UNG kit and Taqman miRNA Assays (20X primers; hsa‐miR‐1, ‐16, ‐23b and ‐133a/b; Life Technologies). The PCR conditions consisted of 10 min at 95°C; 40 cycles of 15 s at 95°C, and 60 s at 60°C. All samples were run in triplicate, with an internal control included on successive plates for individual miRNAs.

Skeletal muscle miRNA expression was normalized to the median of U6 and RNU48 and calculated using the 2^−ΔΔCq^ method. As there is currently no known stable endogenous control suitable for normalizing EV miRNA expression (D'Souza et al., 2018; Lovett, Durcan, & Myburgh, 2018), EV miRNA expression was expressed relative to the total RNA input and calculated using the 2^−ΔCq^ method. In EV RNA, spike‐in cel‐miR‐39 was stably expressed across all time points, indicating efficient and reproducible reverse transcription and qPCR processes.

### 2.11 Sample size

To date, no studies have investigated changes in either skeletal muscle or EV miRNA expression in response to exercise in a female cohort. However, testosterone and oestrogen both positively regulate miR‐133a in vitro (Huang, Wong, Seto, Lai, & Yuen, 2015; Nielsen, Hvid, et al., 2014). It is therefore feasible that male and female sex hormones positively regulate miR‐133a, and other skeletal muscle‐enriched miRNAs, to a similar extent in human models. Thus, this study was designed to include both males and females as a homogeneous cohort. Using data obtained previously by our group investigating the effect of an identical exercise protocol on skeletal muscle miRNA expression in males (Russell et al., 2013), power analyses (β = 0.20, α = 0.025 (to correct for multiple time points), one‐tailed) indicated that to detect an 80% increase in miR‐1 expression (calculated using pre and 3 hr post exercise values of 1.0 ± 0.78 and 1.8 ± 1.20, respectively; mean ± *SD*; Russell et al., 2013), a sample size of 15 participants was sufficient.

### 2.12 Statistical analyses

All statistical analyses were conducted using GraphPad Prism (v8.0; CA, USA). To test whether EV miRNA expression is a proxy for skeletal muscle miRNA expression, correlations between skeletal muscle and EV miRNA expression were analysed using Pearson's correlation coefficient. A one‐way, repeated measures analysis of variance (ANOVA) was used to identify significant differences in EV and skeletal muscle miRNA expression in response to endurance exercise, with Tukey post hoc analysis where appropriate. All data were first analysed as a homogenous cohort, including all male and female participants. Secondary analysis to explore possible sex‐specific differences in EV and skeletal muscle miRNA expression in response to exercise was then performed. Caution should be used when interpreting these exploratory results, as this study was not originally designed nor powered to look at sex‐specific differences. Unless otherwise indicated, all data are represented as mean ± *SD*. Statistical significance was set at *p* < .05.

## 3 RESULTS

### 3.1 Participant characteristics

Participant anthropometrics are summarized in Table 1. No significant differences were observed between males and females for age, BMI or relative VO_2_peak. All participants completed the 60 min bout of acute cycling at approximately 70% VO_2_peak.

### 3.2 EV RNA yield

RNA extracted from 1 ml of plasma resulted in total RNA yields of 8.89 ± 5.44 ng, 9.34 ± 7.15 ng and 6.48 ± 5.06 ng (mean ± *SD*) at rest, immediately post exercise and 3 hr post exercise, respectively. Total RNA yield per ml plasma was significantly less 3 hr postexercise compared to pre and immediately post exercise. However, hydration status of the participants was not controlled. Therefore, the changes in total RNA concentration may be an artifact of fluctuations in plasma volume. This constitutes a limitation of this study.

### 3.3 Skeletal muscle and EV miRNA expression with exercise

**Figure 2:**
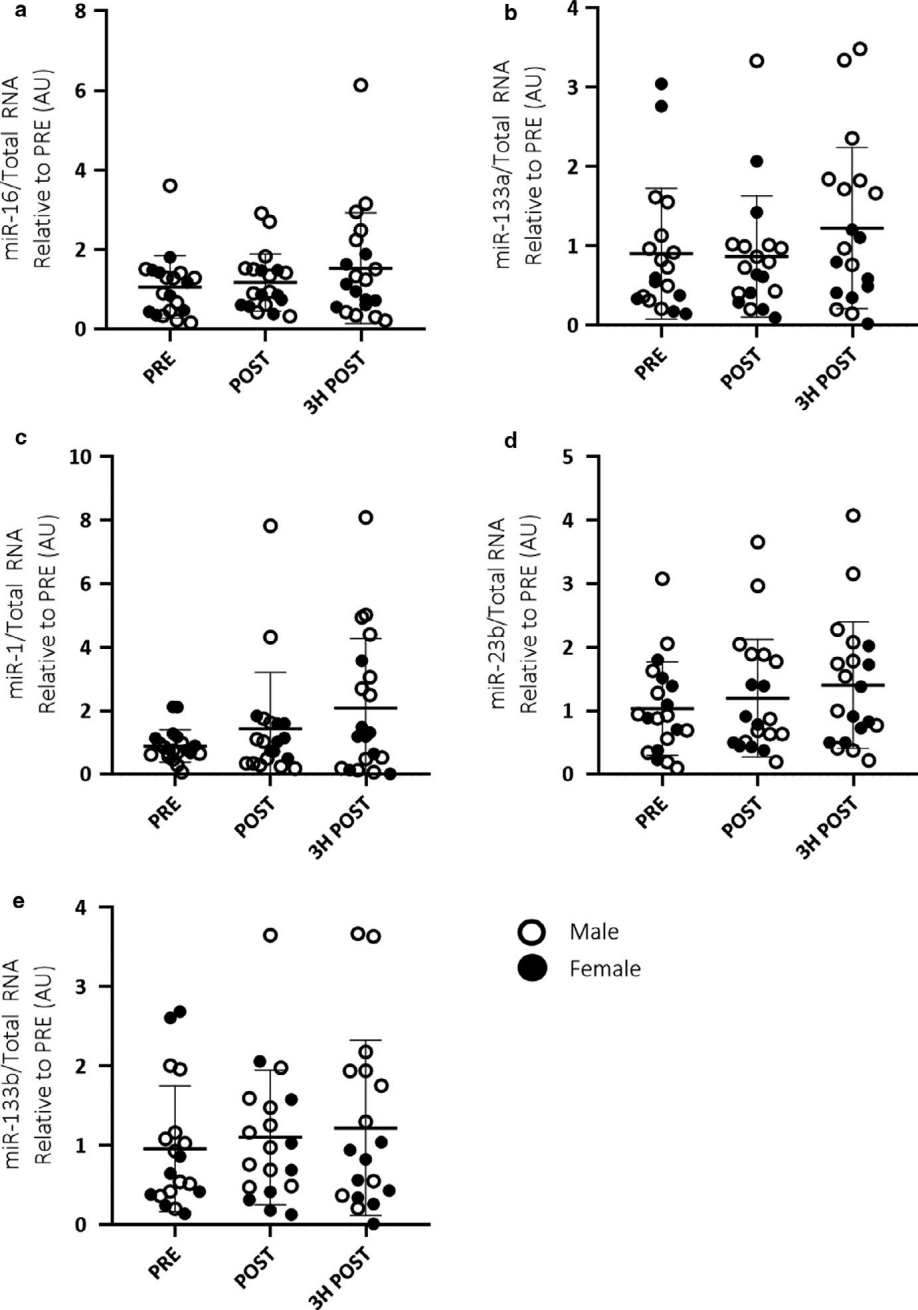
Combined male (*n* = 12) and female (*n* = 8) EV miRNA expression (miR‐16, ‐133a, ‐1, ‐23b and ‐133b; a–e, respectively) in response to 60 min of cycling at 70% VO_2_peak. A one‐way, repeated measures ANOVA was used to identify significant differences in miRNA expression over time. Individual data points shown for each participant along with mean ± *SD*

**Figure 3:**
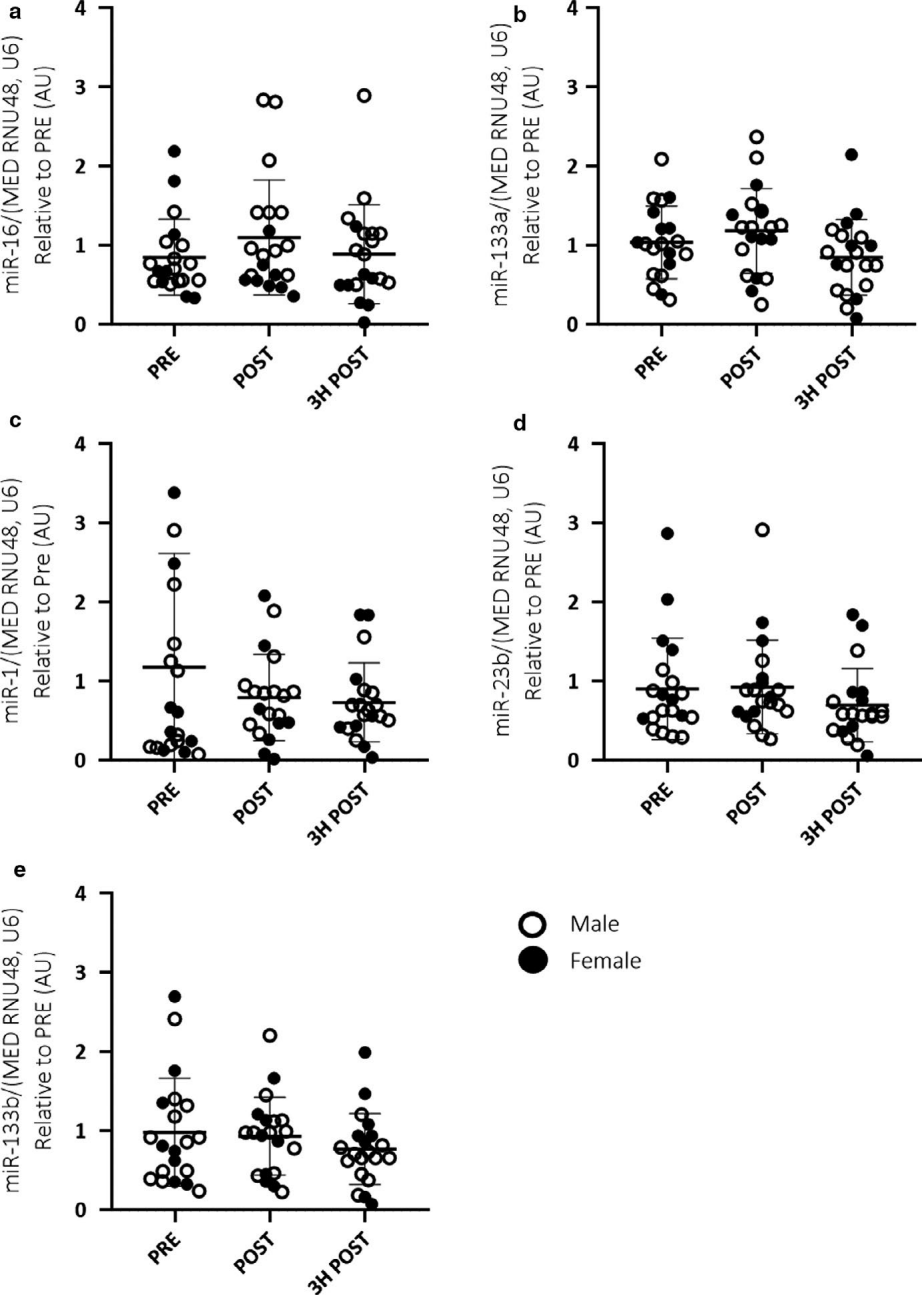
Combined male (*n* = 12) and female (*n* = 8) skeletal muscle miRNA expression (miR‐16, ‐133a, ‐1, ‐23b and ‐133b; a–e, respectively) in response to 60 min of cycling at 70% VO_2_peak. A one‐way, repeated measures ANOVA was used to identify significant differences in miRNA expression over time. Individual data points shown for each participant along with mean ± *SD*

**Figure 4:**
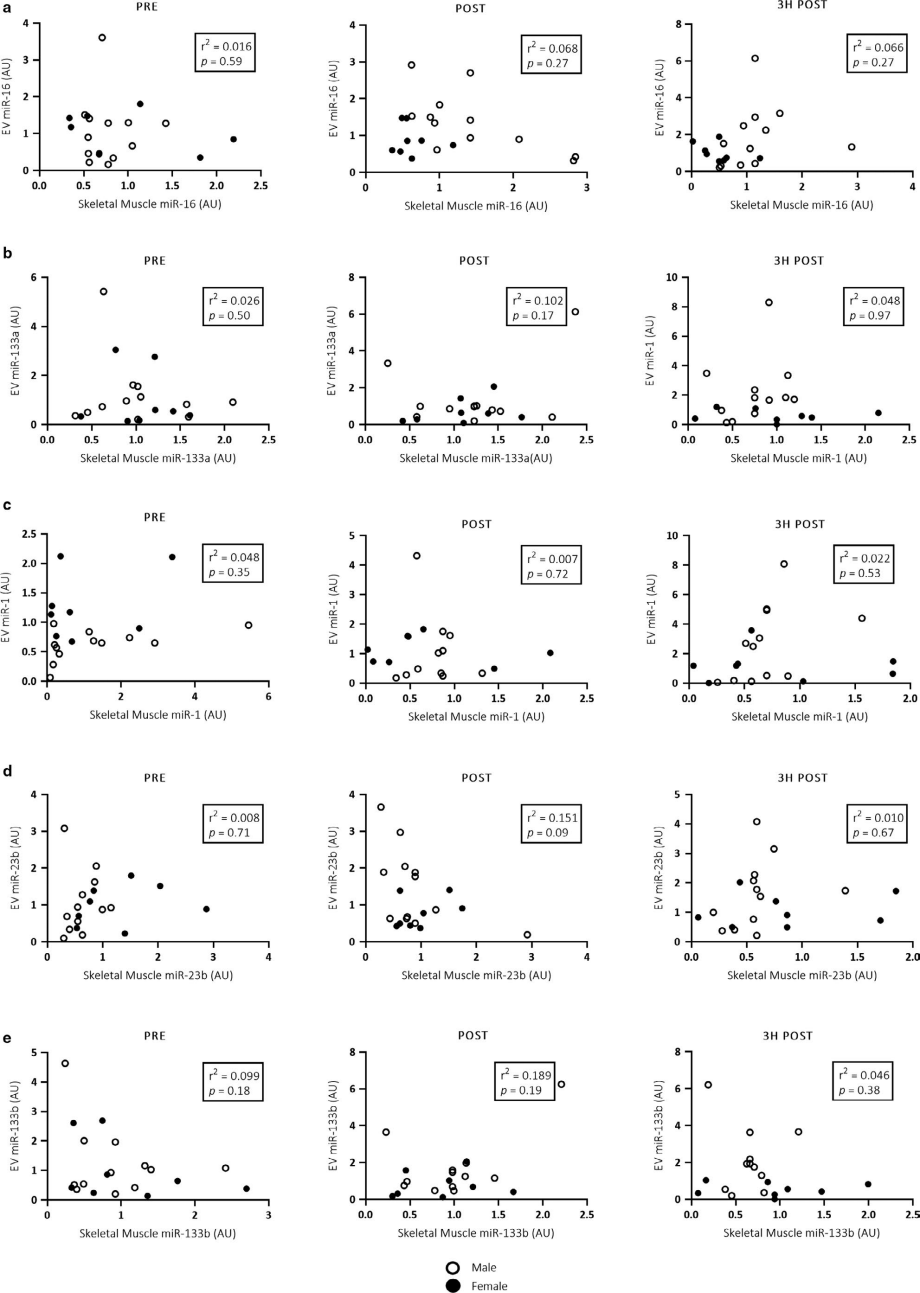
Pearson correlations between EV and skeletal muscle miR‐16, ‐133a, ‐1, ‐23b and ‐133b (a–e, respectively) expression in the combined male (*n* = 12) and female (*n* = 8) cohort pre, immediately post and 3 hr post a single bout of cycling at 70% VO_2_peak. No significant correlations between EV and skeletal muscle miRNA expression were observed

When analysed as a homogenous cohort combining both males and females, no significant differences were observed in either EV (Figure 2) or skeletal muscle (Figure 3) miRNA expression in response to acute endurance exercise. Additionally, there were no significant correlations between EV and skeletal muscle miRNA expression (Figure 4).

### 3.4 Sex‐specific skeletal muscle miRNA expression with exercise

**Figure 5:**
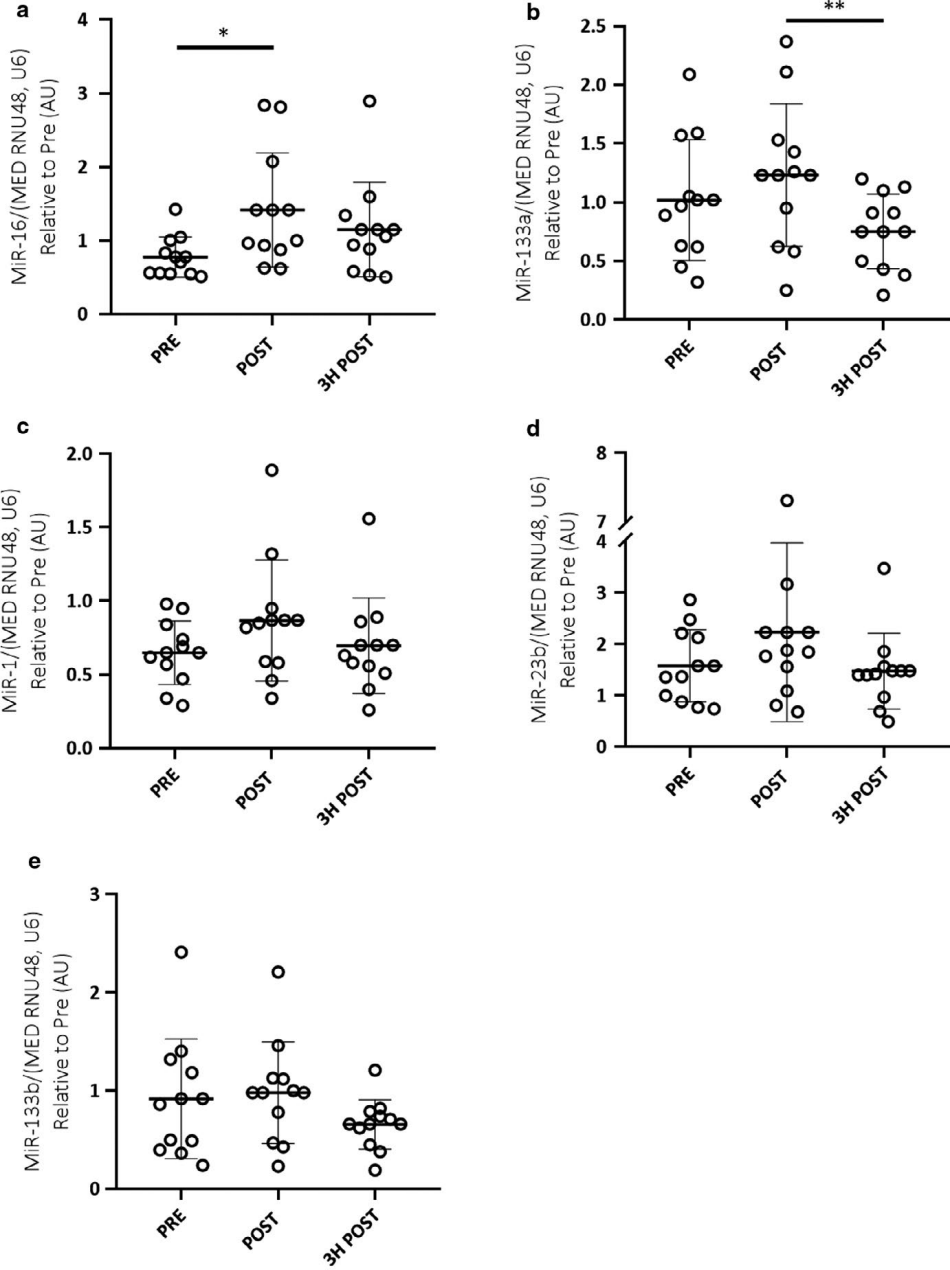
Male skeletal muscle (*n = *12) miRNA expression (miR‐16, ‐133a, ‐1, ‐23b and ‐133b; a–e respectively) in response to 60 min of cycling at 70% VO_2_peak. A one‐way, repeated measures ANOVA was used to identify significant differences in miRNA expression over time. Individual data points shown for each participant along with mean ± *SD*. *p < .05; **p < .01

When sexes were analyzed separately, male skeletal muscle miR‐16 (*p* < .05; Figure 5a) expression significantly increased immediately following exercise when compared with pre values. MiR‐133a (*p* < .01; Figure 5b) expression significantly decreased 3 hr post exercise when compared to immediately postexercise. No significant differences were observed in miR‐1 (Figure 5c), miR‐23b (Figure 5d) and miR‐133b (Figure 5e) expression in response to exercise.

No significant differences were observed in female skeletal muscle miRNA expression (miR‐1, miR‐16, miR‐23b, miR‐133a/b) in response to acute endurance exercise.

### 3.5 Sex‐specific correlations between EV and skeletal muscle miRNA expression

**Figure 6:**
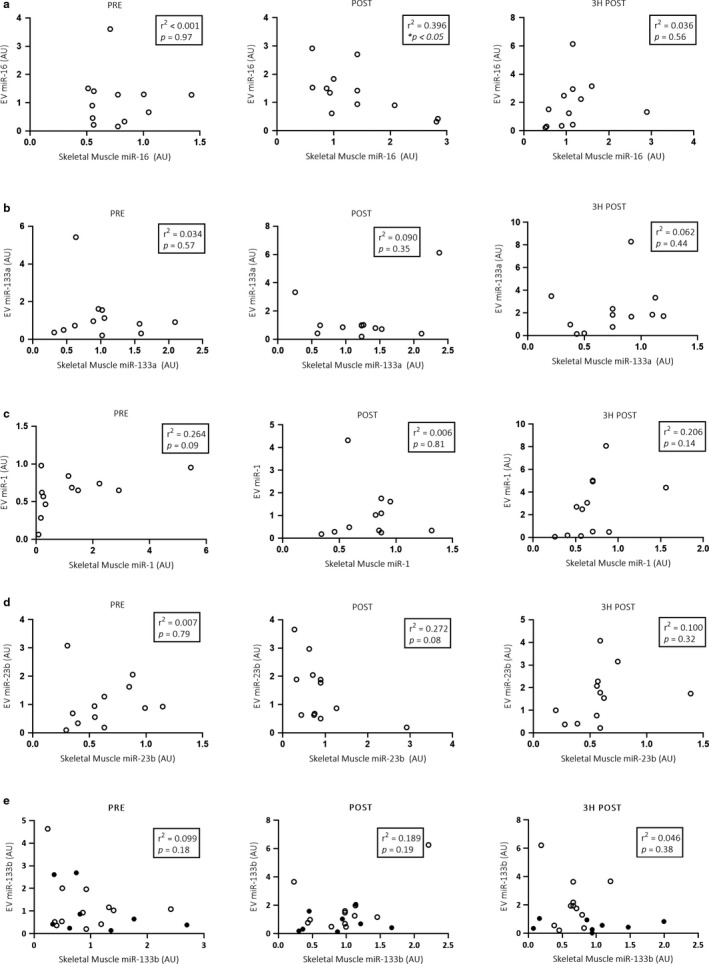
Pearson correlations between EV and skeletal muscle miR‐16, ‐133a, ‐1, ‐23b and ‐133b (a–e, respectively) expression in the male (*n* = 12) cohort pre, immediately post and 3 hr post a single bout of cycling at 70% VO_2_peak. *p < .05

**Figure 7:**
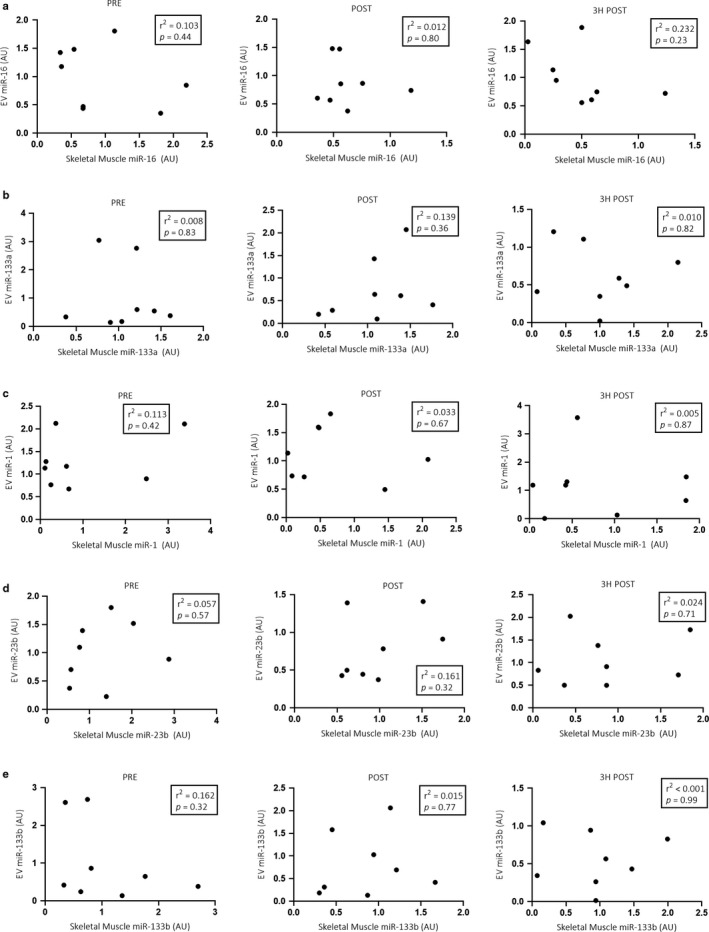
Pearson correlations between EV and skeletal muscle miR‐16, ‐133a, ‐1, ‐23b and ‐133b (a–e, respectively) expression in the female (*n* = 8) cohort pre, immediately post and 3 hr post a single bout of cycling at 70% VO_2_peak. No significant correlations between female EV and skeletal muscle miRNA expression were observed

When sexes were analyzed separately, a moderate negative correlation was observed between EV and skeletal muscle miR‐16 expression (*p* < .05; *r* ^2^ = 0.396) immediately post exercise in males (Figure 6a), but not females (Figure 7). No further correlations between EV and skeletal muscle miRNA expression were observed pre, post or 3 hr post exercise in either males or females (Figures 6 and 7).

## 4 DISCUSSION

This study primarily aimed to describe EV miRNA expression in a cohort of both males and females after an acute bout of continuous, moderate intensity cycling. We observed no differences in miR‐1, ‐16, ‐23b, ‐133a/b expression in EVs or skeletal muscle after exercise. Furthermore, we observed no correlation between EV and skeletal muscle miRNA expression, suggesting that EV miRNA expression is not a proxy for skeletal muscle miRNA expression. When the cohort was split into males and females for exploratory analysis, skeletal muscle miR‐16 expression significantly increased immediately post exercise while miR‐133a significantly decreased 3 hr post exercise in males, but not females. This presents the first known evidence of differential miRNA responses following endurance exercise between sexes in vivo.

### 4.1 EV miRNA expression following acute endurance exercise

The field of EV miRNA content in response to exercise is a relatively new area of research. This study observed no change in EV miRNA expression following a single bout of moderate intensity, continuous exercise. These findings are in agreement with previous research that examined EV miR‐1, ‐133a/b, ‐181a, ‐206, ‐499 and ‐146a before and one hour after a bout of continuous, moderate intensity (65% VO_2_peak) running exercise in healthy young men (*n* = 7; Guescini et al., 2015). Of these species, only miR‐181a was significantly upregulated after exercise compared to miR‐16 or exogenous spike‐in cel‐miR‐39 (Guescini et al., 2015). These findings contrast with a recent paper that observed increases in EV miR‐1, ‐16, ‐21, ‐23a/b, ‐107, ‐126, ‐134, ‐150, ‐186, ‐208a, ‐222, ‐378a, ‐486 and ‐451a after HIIE in young, healthy men (*n* = 10; D'Souza et al., 2018).

The contrasting findings may be due to the impact of differing exercise intensities on EV yields (Frühbeis et al., 2015). For example, EV release and cargo are known to be stimulated by decreases in pH to 6.0, albeit in melanoma cells in vitro (Li et al., 2017; Parolini et al., 2009). In human models, skeletal muscle pH is known to remain between 7.08 and 7.16 during submaximal cycling (70% VO_2_peak) followed by 60 min of passive recovery (Booth et al., 1997). In contrast, maximal exercise reduces muscular pH as low as 6.42 (Hermansen & Osnes, 1972), and femoral blood pH to 6.96 (Sahlin, Alvestrand, Brandt, & Hultman, 1978). While this decrease in pH is not as low as shown in in vitro models, there is evidence that maximal intensity and intermittent exercise decreases plasma and muscle pH to a larger extent compared to moderate intensity, continuous exercise (Cairns, 2006). To this end, further research is now required into the impact of exercise intensity on EV miRNA expression.

Different miRNA normalization strategies may further explain the contrasting results between studies. Currently, there is no consensus on the most appropriate normalization strategy for EV miRNA normalization within the scientific community (Schwarzenbach, da Silva, Calin, & Pantel, 2015). This study used a standardized amount of EV RNA (500 pg) per reaction. In contrast, previous research (D'Souza et al., 2018; Guescini et al., 2015) normalized circulatory and EV miRNA expression to total RNA per ml of plasma. Although normalizing EV miRNA expression to total RNA per ml of plasma may provide a more physiological representation of the total EV miRNA pool released into the circulation during exercise, plasma volume decreases by up to 13% with continuous exercise (Novosadová, 1977), and would thus overestimate EV miRNA levels by at least 13%. We therefore suggest that normalizing EV miRNA expression based on plasma volume may not be the most appropriate normalization strategy. Another normalization strategy used in previous human EV miRNA studies is to normalize to miR‐16 (Guescini et al., 2015). However, this approach, does not appear suitable given EV miR‐16 expression can increase up to 10‐fold following HIIE (D'Souza et al., 2018). Although EV miR‐16 was not differentially expressed in response to exercise in this study, we did observe large individual variation over time. Our findings in this study and those of D'Souza et al. (D'Souza et al., 2018) suggest that miR‐16 should not be used for normalizing EV miRNA expression.

While we cannot comment on EV miRNA yield following exercise, total RNA yield was significantly lower 3 hr postexercise, compared to pre or immediately post exercise. This could be suggestive of less EVs being released 3 hr after exercise, or it could indicate that less RNA is released within each EV. However, as plasma volume was not measured nor controlled for, this may also simply be a result of fluctuations in plasma volume, rather than a physiological change in RNA or EV release. This is an interesting area for future research, however, these findings should be interpreted with caution.

### 4.2 Skeletal muscle miRNA expression following acute endurance exercise

In males, this study observed an increase in skeletal muscle miR‐16 expression immediately post exercise, whilst miR‐133a expression decreased during recovery (3 hr post). Despite recruiting participants of comparable age and aerobic fitness levels, these results are in contrast to similar research in this field (Nielsen et al., 2010; Russell et al., 2013). Nielsen et al. (2010) observed increases in male skeletal muscle miR‐1 and miR‐133a expression immediately following 60 min of cycling at 65% maximal power output (P_max_), returning to baseline levels following 3 hr recovery (Nielsen et al., 2010). In contrast, we have previously observed increased miR‐1, miR‐133a/b expression, and decreased miR‐23b expression 3 hr post exercise when compared with resting values (Russell et al., 2013).

As with research into differential miRNA expression in EVs, normalization strategies may partially account for the contrasting results skeletal muscle miRNA expression observed between groups. Skeletal muscle miRNA expression is routinely normalized to RNU48 or U6 in both human (Nielsen et al., 2010; Russell et al., 2013) and animal (Safdar et al., 2009; Xu, Zhao, Sun, Liu, & Zhang, 2015) exercise models. However, it has since been suggested that using the median of multiple (up to six) house‐keeping genes (Pinto et al., 2017) may be a superior approach for miRNA normalization. Thus, this study normalized skeletal muscle miRNA expression to the median of RNU48 and U6. However, the use of different normalization methods between studies may limit our ability to make direct comparisons to previous research in this field (Nielsen et al., 2010; Russell et al., 2013). Relatively small changes in miRNA expression may be physiologically significant, particularly if it induces changes in downstream protein expression and the subsequent adaptive responses to exercise (Meyer, Pfaffl, & Ulbrich, 2010). It is therefore important to consider appropriate endogenous controls that minimize individual variability, and thus are more likely to reflect true physiological differences in miRNA expression in response to exercise.

### 4.3 Sex differences in male and female skeletal muscle miRNA expression

Males comprise an overwhelming majority of participants in exercise physiology and sport research (Knowles et al., 2019), and are a historically convenient target population for exercise research. Recently it was estimated that only 4%–13% of research in top ranked sports science journals included female‐only cohorts (Costello, Bieuzen, & Bleakley, 2014). To date, skeletal muscle and EV miRNA expression in response to exercise has been investigated in solely male cohorts (D'Souza et al., 2018; Nielsen et al., 2010; Russell et al., 2013). Although the sex‐specific differences observed in this study are exploratory and likely underpowered, our data present a novel aspect that may begin to elucidate sex‐specific mechanisms by which skeletal muscle and whole‐body adaptations are regulated in response to endurance exercise. Contrary to our assumptions, our exploratory data show differential responses to exercise in the skeletal muscle miRNA profile between males and females. We observed males to have a significant increase in miR‐16 immediately post exercise, and significant decreases in miR‐133a 3 hr post exercise, while females did not. While this should be interpreted with caution, it is the first evidence to suggest a potential sex‐specific miRNA response to exercise. This reinforces the need to further examine the ncRNA response to endurance exercise in respective male and female cohorts in vivo.

### 4.4 Correlations between EV and skeletal muscle miRNA expression

A weak negative correlation was observed between skeletal muscle and EV miR‐16 expression immediately following the acute bout of exercise in males, although the physiological relevance of the divergent miR‐16 responses following endurance exercise in skeletal muscle and EVs is unclear. MiR‐16 is implicated in the regulation of angiogenesis (Fernandes, Magalhaes, Roque, Phillips, & Oliveira, 1979). We might therefore anticipate increases in EV miR‐16 expression following endurance exercise, thus presenting one mechanism by which muscular adaptations are conferred to other tissues (i.e., the vascular network). However, this was not observed in this study. The role of miR‐16 in skeletal muscle exercise adaptations is not well understood and may be a target for future research. The lack of correlation between skeletal muscle and EV miRNA expression for all other miRNA species investigated in this study corroborates previous evidence stemming from a single bout of HIIE (D'Souza et al., 2018). This suggests that EV miRNA expression is not a proxy for skeletal muscle miRNA expression at rest or in response to acute endurance exercise, and therefore reinforces the need for tissue‐specific miRNA expression analysis.

Studies investigating possible correlations between EV and skeletal muscle miRNA expression must consider the origin of the miRNAs. MiRNAs packaged within EVs may be secreted from numerous tissues throughout the body, and thus, we cannot be sure that the miRNAs detected within EVs were secreted from the vastus lateralis. Furthermore, the location of muscle biopsies and blood samples must be taken into account. Protocols using in‐dwelling cannulas in the femoral artery and vein increase the probability that any isolated EV were directly released from the contracting muscles (Whitham et al., 2018). Although the blood sampling method used in this study is replicated throughout the literature (D'Souza et al., 2018; Frühbeis et al., 2015; Lovett et al., 2018), the limitation of this study, and ultimately many in this field, is that blood samples and muscle biopsies were taken from different parts of the body. By examining muscle‐enriched miRNA species (Silver, Wadley, & Lamon, 2018), the likelihood that the miRNAs examined were released by skeletal muscle is increased; however, these species are also enriched in cardiac muscle. It must be recognized that this is a limitation of this study and we cannot conclusively determine the origin of the isolated EVs.

## 5 CONCLUDING REMARKS

Endurance exercise not only promotes local adaptations within skeletal muscle, but also systemic adaptations throughout many organ systems in the human body. EV cargo, such as miRNAs, are postulated to mediate systemic cross‐talk in response to exercise. Despite this hypothesis, no correlations were observed between EV and skeletal muscle miRNA expression in a combined male and female cohort, suggesting that EV miRNA expression is not a proxy for skeletal muscle miRNA expression. Our exploratory analysis identified a weak negative correlation between only EV and skeletal muscle miR‐16 expression immediately post exercise in males, but not females. However, the physiological relevance of this aforementioned correlation is unclear. Furthermore, skeletal muscle miR‐16 expression increased immediately post exercise, whilst miR‐133a expression decreased 3 hr following acute endurance exercise in males, but not in females. Overwhelmingly, much of research in the exercise physiology domain is conducted solely in males; this study provides further evidence that knowledge of male physiology cannot always be inferred to female physiology, nor can miRNA expression in one compartment (i.e., skeletal muscle) be compared to miRNAs packaged within EVs in plasma. We recommended that future studies investigating physiological responses to endurance exercise, including changes in miRNA expression, concurrently investigate comparable male and female cohorts to elucidate the sex‐specific differences in the miRNA and EV response to both continuous and high intensity exercise.

## DISCLOSURE

The authors have nothing to disclose.

## AUTHOR CONTRIBUTION

G.D.W. is the guarantor of this work and, as such, had full access to all the data in the study and takes responsibility for the integrity of the data and the accuracy of the data analysis. All authors performed the experiments, were involved in the data analysis and drafting of the manuscript. All authors critically revised and approved the manuscript.
