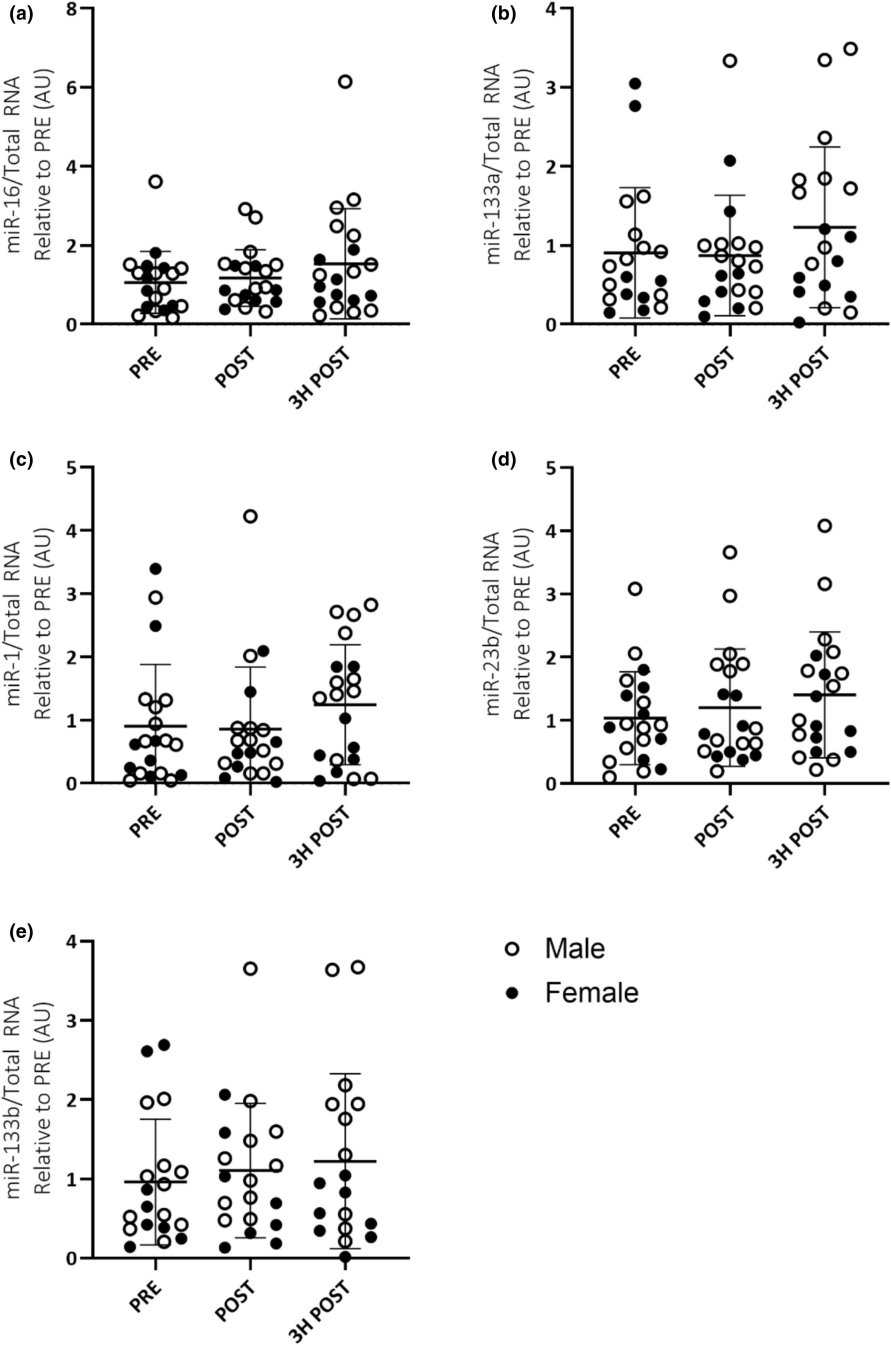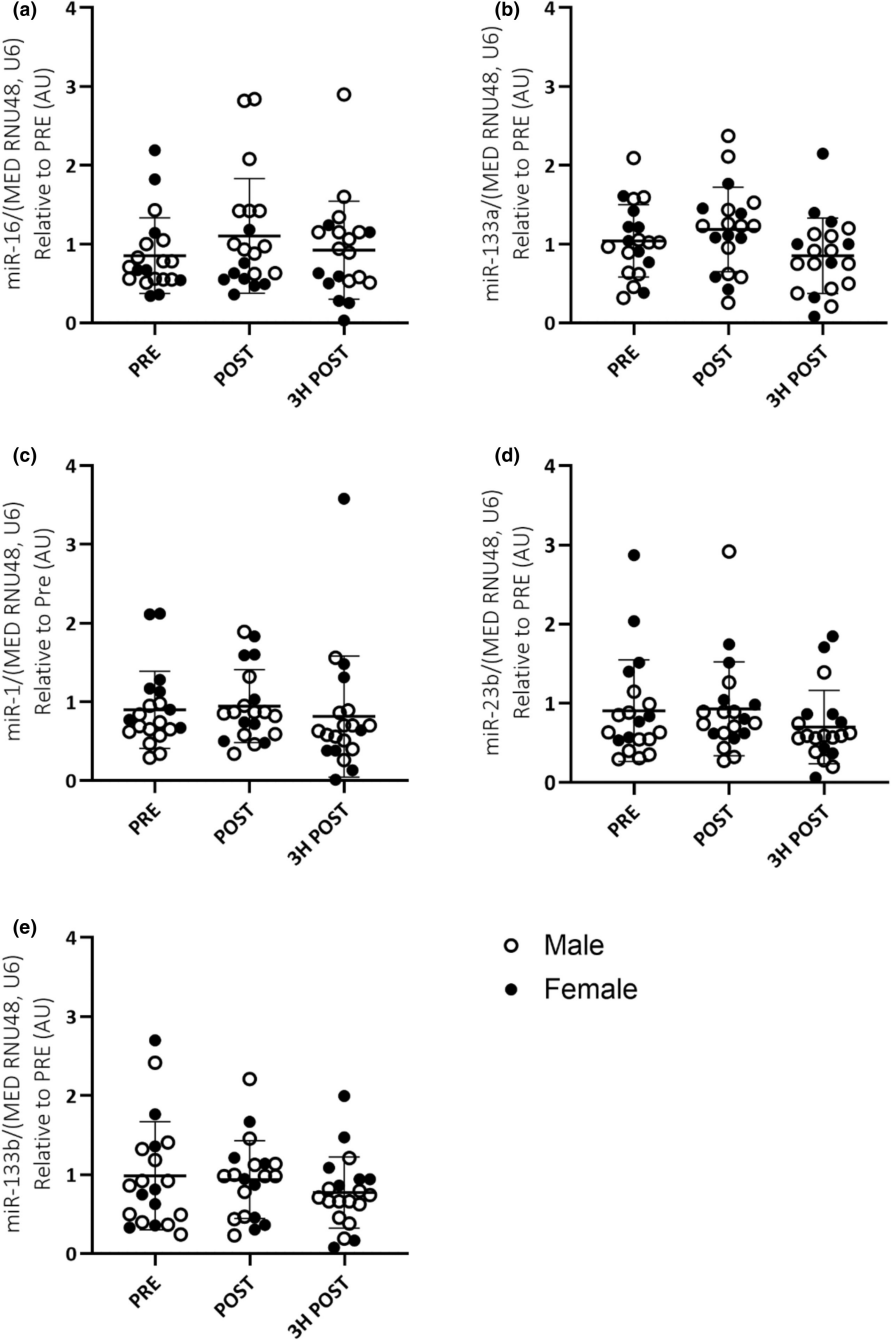# Correction to “Extracellular vesicular miRNA expression is not a proxy for skeletal muscle miRNA expression in males and females following acute, moderate intensity exercise”

**DOI:** 10.14814/phy2.16163

**Published:** 2024-07-22

Silver J.L., S.E. Alexander, H.T. Dillon, S. Lamon, and G.D. Wadley. Physiological Reports. 2020;8:e14520.

A recent audit of our source data for the manuscript discovered an error in our collated data that has resulted in the raw data in Figure 2C and Figure 3C (miR‐1 expression in EVs and skeletal muscle, respectively) being displayed incorrectly. The correct data are shown in the updated versions of Figure 2C and Figure 3C provided here. This has not altered the findings of the study or the interpretation of the data.

We apologize for this error.